# Application of the Multidimensional Fatigue Inventory to Ethiopian Cancer Patients

**DOI:** 10.3389/fpsyg.2021.687994

**Published:** 2021-12-02

**Authors:** Yemataw Wondie, Andreas Hinz

**Affiliations:** ^1^Department of Psychology, University of Gondar, Gondar, Ethiopia; ^2^Department of Medical Psychology and Medical Sociology, University of Leipzig, Leipzig, Germany

**Keywords:** cancer-related fatigue, quality of life, reliability, cancer, illiteracy

## Abstract

**Objectives:** Fatigue is a frequent debilitating symptom associated with cancer. However, scientific data on cancer-related fatigue is scarce in developing nations. This work examines psychometric properties of the multidimensional fatigue inventory (MFI-20) and analyzes the level of fatigue among Ethiopian patients with cancer in comparison with data from Germany.

**Methods:** A sample of 256 patients with cancer drawn from a hospital in Ethiopia was examined with the MFI-20 and the European Organization for Research and Treatment of Cancer Quality of Life questionnaire (EORTC QLQ-C30). A comparative sample of 780 German patients with cancer served as the control.

**Results:** The MFI-20 scales and total score showed acceptable reliability (α = 0.60–0.93) with a considerable convergent validity between MFI-20 and the EORTC QLQ-C30 fatigue scale (*r* = 0.67–0.75). The Ethiopian patients with cancer reported higher levels of fatigue than the German patients. Analyses of variance showed that Ethiopian patients with cancer who were illiterate, having advanced cancer, and those who did not receive either surgery or chemotherapy reported especially high levels of fatigue.

**Conclusion:** The MFI-20 is a fairly reliable and valid instrument to be used with Amharic speaking patients with cancer. The high level of fatigue in these patients implies that appropriate cancer care is needed in developing countries.

## Introduction

Being diagnosed with cancer drastically affects one’s physical and mental states. Patients with cancer experience a range of symptoms that affect their quality of life (QoL). These symptoms have been studied extensively in high-income countries (HIC) where cancer care resources are accessible. However, little is known regarding the mental health consequences and QoL of populations with cancer residing in low and middle-income countries (LMIC), including Africa, where resources are limited (Muliira et al., 2017). More specifically, annual number of new cases of cancer in Africa is estimated to grow to more than one million in the next 5 years, which is associated with immense loss in human life and considerable economic setback (Boyle et al., 2019). The WHO reported that about 70% of the world’s cancer-related deaths are from LMICs alone (World Health Organization, 2015). Cancer specialists working in Africa and international experts working closely with African colleagues portray the relationship between cancer and resource limitations as “It is bad to have cancer and worse to have cancer if you are poor” (Boyle et al., 2019). This can be demonstrated by premature death of cancer where the rate is only two out of five in HICs, whereas it may raise to nine out of 10 people in resource-poor countries (Boyle et al., 2019). All these facts show that cancer has become a major health problem in Africa (Boyle et al., 2016).

Patients living with or being treated for cancer report a wide range of side effects and symptoms some of which emanate from the disease itself, the treatments received, or the patient’s emotional state (Wagland et al., 2015). Fatigue is one of the most pressing burdens frequently reported by patients with cancer (Prue et al., 2006; Berger et al., 2015). In the context of cancer, fatigue is defined as a distressing, persistent, subjective sense of physical, emotional, and/or cognitive tiredness or exhaustion related to cancer or cancer treatment that is not proportional to recent activity and interferes with usual functioning (Berger et al., 2010). Fatigue in patients with cancer is also described as a common and debilitating burden (Dash et al., 2016). Depending on the measures used and the cancer stages, the prevalence of fatigue in patients with cancer ranges from 60 to 99% (Lawrence et al., 2004). On the other hand, clinicians from LMICs view fatigue as an underassessed and underreported aspect of cancer patient’s treatment (Parveen et al., 2019). Behavioral intervention techniques to reduce the level of fatigue include physical exercise, muscle relaxation, and meditation (Andersen et al., 2013; Metin et al., 2019).

A number of unidimensional and multidimensional instruments have been used to measure the level of fatigue among patients with cancer and other diseases (Al Maqbali et al., 2019). One of the frequently used instruments is the multidimensional fatigue inventory, (MFI-20) which is characterized by general fatigue, physical fatigue, reduced activity, reduced motivation, and mental fatigue dimensions (Smets et al., 1995). The MFI-20 has been translated into several languages including French (Fillion et al., 2003), Chinese (Tian and Hong, 2012), Brazilian Portuguese (Baptista et al., 2012), Polish (Buss et al., 2014), Hindi (Chandel et al., 2015), and Swedish (Hagelin et al., 2007) since its original validation in Dutch patients with cancer and non-cancer (Smets et al., 1995). Acceptable reliability coefficients were reported in all MFI-20 versions ranging from 0.80 to 0.90 (Al Maqbali et al., 2019).

A few works have been conducted on QoL (Tadele, 2015; Ayana et al., 2018; Araya et al., 2019; Wondimagegnehu et al., 2019) and fatigue (Gebremariam et al., 2018; Sibhat et al., 2019) in Ethiopian patients with cancer. Most of these works were conducted in health facilities of Addis Ababa, the capital city of Ethiopia. To the best of our knowledge, only one of the works (Gebremariam et al., 2018) performed in Ethiopia so far reported the level of fatigue, using the brief fatigue inventory.

The specific objectives of this study were: (a) to examine the level of fatigue in Ethiopian patients with cancer in comparison with their German counterparts, (b) to measure the impact of socio-demographic and clinical characteristics on fatigue, (c) to analyze the relationship between the MFI-20 dimensions and total score with the EORTC QLQ-C30 dimensions and total score, and (d) to determine the psychometric properties of the MFI-20.

## Methods

### Ethiopian Patients With Cancer

This study was conducted at the University of Gondar Hospital, Ethiopia. Two hundred and ninety eight patients with cancer treated between January 2018 and April 2019 at this hospital were eligible for this study. The inclusion criteria being: patients with a diagnosed malignant tumor, being at least 18 years old, and understanding Amharic, the official language of the country and predominantly spoken in Gondar and its surroundings. Tumor entities, disease stage, and illiteracy did not serve as exclusion criteria. We recruited two oncology nurses as research assistants. Adequate training was given to them on the Amharic versions of the questionnaires. The research assistants contacted the patients, explained the aims of the study, and asked them to participate and give informed consent. If the patients were illiterate, the research assistants read the questions aloud, asked for verbal responses, and marked the responses in the questionnaire. In most cases the questionnaires were filled in before the beginning of the treatment. Medical data was taken from the medical records of the hospital. The study was conducted in accordance with the Declaration of Helsinki and was approved by the Institutional Ethical Review Board of the University of Gondar (Ref. No. O/V/P/RCS/05/1542/2018; dated June 18, 2018).

### German Patients With Cancer

The control group of this work was German patients with cancer selected from several cancer centers in Germany. Methodological details and sample characteristics have already been published (Mehnert et al., 2012). The sample comprised of 1,821 men and 1,964 women with a mean age of 58.3 years. To match with the Ethiopian sample in age and gender distribution, we selected a subsample of the German patients with cancer. The matched German subsample consisted of 780 patients with cancer of which 305 (39.1%) and 475 (60.9%) were men and women, respectively. The mean age of this group was 47.9 years. The German study was also conducted in accordance with the Declaration of Helsinki and was approved by the Ethics Committees of each of the five participating universities.

### Instruments

#### Multidimensional Fatigue Inventory

The MFI-20 is composed of 20 items which belong to one of the five dimensions (general fatigue, physical fatigue, reduced activity, reduced motivation, and mental fatigue). The responses of each item are arranged in a Likert scale ranging from 1 (Yes, this is true) to 5 (No, this is not true). The scores of each dimension range from 4 to 20. Each of the dimensions contains two positively and two negatively orientated items. It is possible to use a sum score of the 20 items (Kuhnt et al., 2009).

#### European Organization for Research and Treatment of Cancer Quality of Life Questionnaire

The EORTC QLQ-C30 (Aaronson et al., 1993) consists of 30 items categorized under five functioning scales (physical, role, emotional, social, and cognitive functioning), three symptom scales (fatigue, pain, and nausea/vomiting), a two-item global health/QoL scale, and six single-item scales (dyspnea, appetite loss, insomnia, constipation, diarrhea, and financial difficulties). While higher functioning scores represent better functioning/QOL, higher symptom scores denote more severe symptoms. The EORTC QoL Group (Giesinger et al., 2016) recommends a sum score of the EORTC QLQ-C30 that consists of the scores of the functioning scales and the inverted scores of the symptom scores. This means that high sum scores indicate a high level of QoL.

### Statistical Analysis

Cohen’s effect size *d* was employed to measure mean score differences. Cronbach’s coefficient alpha was used to measure reliability. We used the three-factor analyses of variance (ANOVAs) for testing the impact of socio-demographic and clinical variables on fatigue with gender and age group as cofactors. The associations between the MFI-20 scales and dimensions of the EORTC QLQ-C30 were measured using Pearson correlation coefficients. All the statistics were calculated with SPSS version 24.

## Results

### Socio-Demographic and Clinical Characteristics of the Sample

Table 1 portrays socio-demographic and clinical characteristics of the Ethiopian patients with cancer. Of the 298 patients with cancer illegible for the study, 256 (86%) responded to the questionnaire. The mean age was 47.9 years; 99 (38.7%) were men and 157 (61.3%) were women. More than half (135) were illiterate in which case the nurse research assistants read the items aloud and marked the patients’ responses.

**TABLE 1:** Characteristics of the sample of Ethiopian cancer patients.

	Males		Females		Total	
	(*n* = 99)		(*n* = 157)		(*n* = 256)	
	*n*	%	*n*	%	*n*	%
**Age (years)**						
*M* (SD)	51.6	(15.8)	45.6	(13.3)	47.9	(14.6)
**Age category**						
18–49 years	37	37.4	90	57.3	127	49.6
≥50 years	62	62.6	67	42.7	129	50.4
**Marital status**						
Single	16	16.2	23	14.6	39	15.2
Married	76	76.8	82	52.2	158	61.7
Divorced	7	7.1	30	19.1	37	14.5
Separate/Widowed	0	0.0	22	14.0	22	8.6
**Education**						
Illiterate	48	48.5	87	55.4	135	52.7
Elementary school	19	19.2	19	12.1	38	14.8
Secondary school	13	13.1	18	11.5	31	12.1
Preparatory school	5	5.1	7	4.5	12	4.7
Technical and vocational college	9	9.1	16	10.2	25	9.8
University	5	5.1	10	6.4	15	5.9
**Religion**						
Christian	89	89.9	41	89.8	230	89.8
Muslim	10	10.1	16	10.2	26	10.2
**Tumor**						
Breast	2	2.0	63	40.1	65	25.4
Colon	24	24.2	20	12.7	44	17.2
Non-Hodgkin lymphoma	22	22.2	15	9.6	37	14.5
Cervix uteri	0	0.0	15	9.6	15	5.9
Corpus uteri	0	0.0	9	5.7	9	3.5
Prostate	9	9.1	0	0.0	9	3.5
Colorectal	3	3.0	5	3.2	8	3.1
Thyroid	2	2.0	6	3.8	8	3.1
Lymphocytic lymphoma	2	2.0	4	2.5	6	2.3
Pancreas	5	5.1	1	0.6	6	2.3
Lung	4	4.0	2	1.3	6	2.3
Other	26	26.3	17	10.8	43	16.8
**Tumor stage, UICC^a^**						
1	5	5.1	15	9.6	20	7.8
2	20	20.2	46	29.3	66	25.8
3	22	22.2	41	26.1	63	24.6
4	26	36.4	44	28.0	80	31.3
**Surgery**						
No	56	56.6	69	43.9	125	48.8
Yes	43	43.4	88	56.1	131	51.2
**Radiation**						
No	90	90.9	144	91.7	234	91.4
Yes	9	9.1	13	8.3	22	8.6
**Chemotherapy**						
No	46	46.5	63	40.1	109	42.6
Yes	53	53.5	94	59.9	147	57.4

*M, mean; SD, standard deviation.*

*^a^Missing data not reported.*

### Comparisons of Mean Scores and Reliability Coefficients

Table 2 presents the five dimensions and total score of the MFI-20 and their associated reliability alpha coefficients in comparison between the Ethiopian and the German patients with cancer. The mean differences are expressed in terms of Cohen’s effect size *d*. The highest difference between the Ethiopian group (*M* = 10.5) and German counterparts (*M* = 8.7) was on reduced motivation (*d* = 0.46). There was no significant mean difference between the two groups on the remaining dimensions and total score of the MFI-20. Cronbach’s alpha reliability coefficients were generally good. They ranged from 0.60 to 0.93 in the Ethiopian group and 0.71–0.94 in the German group.

**TABLE 2:** MFI-20 means scores and psychometric criteria, comparison between Ethiopia and Germany.

	Ethiopia		Germany		Effect	Ethiopia	Germany
Scales	*M*	(SD)	*M*	(SD)	size *d*	α	α
General fatigue	12.6	(4.9)	12.5	(4.3)	0.02	0.75	0.84
Physical fatigue	13.0	(5.5)	12.1	(4.7)	0.18	0.79	0.88
Reduced activity	12.5	(4.9)	11.8	(4.6)	0.15	0.79	0.87
Reduced motivation	10.5	(4.2)	8.7	(3.6)	0.46	0.60	0.71
Mental fatigue	10.2	(4.9)	10.1	(4.6)	0.02	0.76	0.88
MFI-20	58.9	(21.5)	55.2	(18.2)	0.19	0.93	0.94

*M, mean; SD, standard deviation; d, effect size of the difference between the Ethiopian and the German mean scores; α, Cronbach’s alpha coefficient.*

### The Impact of Socio-Demographic and Clinical Variables on Fatigue in Ethiopian Patients With Cancer

The impact of socio-demographic characteristics (i.e., gender, age, and education) and clinical variables (i.e., tumor type, tumor stage, and whether surgery or chemotherapy were received) on the scales and total score of MFI-20 was examined. Table 3 describes such associations in the Ethiopian patients with cancer. Older patients with cancer reported a statistically significant higher degree of reduced motivation compared with younger patients (*p* = 0.011). Illiterate patients with cancer demonstrated higher mean scores than literate patients across all the scales and the total score of MFI-20. Except in reduced activity, the differences were statistically significant. Of these, the strongest association between the illiterate and the rest of the groups was on reduced motivation, in which the former reported the highest mean score (*p* < 0.001). Patients with cancer with tumor stage 4 exhibited statistically higher mean scores in all the scales and the total score of the MFI-20. The difference on reduced activity, reduced motivation, mental fatigue, and the total score was statistically significant at *p* < 0.001. Patients with cancer who received surgery showed statistically lower levels of fatigue across the dimensions and the total score, and receiving chemotherapy was associated with significantly lower fatigue levels in all dimensions, except mental fatigue.

**TABLE 3:** MFI mean scores of the Ethiopian sample with socio-demographic and clinical variables.

		General fatigue		Physical fatigue		Reduced activity		Reduced motivation		Mental fatigue		MFI-20 total	
	*n*	*M*	SD	*M*	SD	*M*	SD	*M*	SD	*M*	SD	*M*	SD
**Gender**													
Male	99	13.1	5.1	13.5	5.4	12.9	5.0	10.4	4.2	10.5	4.8	60.4	21.5
Female	157	12.3	4.7	12.7	5.5	12.3	4.9	10.6	4.2	10.0	5.1	57.9	21.5
(Significance)		F = 0.695 p = 0.327		F = 0.585 p = 0.445		F = 0.511 p = 0.475		F = 0.699 p = 0.404		F = 0.287 p = 0.593		F = 2.256 p = 0.107	
**Age group**													
18–49 years	127	11.9	4.7	12.2	5.6	11.8	5.1	9.8	4.1	10.0	5.1	55.8	21.7
≥50 years	129	13.3	5.0	13.7	5.3	13.3	4.7	11.2	4.2	10.4	4.9	62.0	20.8
(Significance)		F = 2.935 p = 0.088		F = 2.789 p = 0.096		F = 3.460 p = 0.064		F = 6.589 p = 0.011		F = 0.373 p = 0.542		F = 0.299 p = 0.585	
**Education**													
Illiterate	135	13.7	4.8	13.8	5.4	13.2	4.9	11.8	3.9	11.0	4.9	63.6	20.0
≤Prep. school	69	11.9	4.8	12.1	5.7	11.9	5.0	9.8	4.1	9.2	4.9	55.1	22.1
College, Univers.	52	10.7	4.6	12.0	5.1	11.4	4.9	8.4	3.9	9.3	4.9	51.9	20.5
(Significance)		F = 9.059 p = 0.003		F = 4.020 p = 0.046		F = 2.318 p = 0.129		F = 15.286 p < 0.001		F = 4.768 p = 0.030		F = 7.949 p = 0.005	
**Tumor type**													
Breast	65	10.9	5.1	11.5	5.5	11.0	6.1	10.0	4.2	9.2	5.3	52.6	23.0
Colon	44	12.3	4.7	12.8	5.3	12.3	4.9	10.5	3.8	9.2	4.1	52.2	19.1
Non-H. lymph.	37	14.0	4.6	13.7	5.1	13.1	4.9	10.7	4.3	11.2	4.8	62.7	20.8
Others	110	13.3	4.6	13.7	5.5	13.3	4.9	10.8	4.3	10.9	5.1	62.1	21.0
(Significance)		F = 2.394 p = 0.069		F = 0.755 p = 0.521		F = 0.479 p = 0.221		F = 0.011 p = 0.998		F = 1.428 p = 0.235		F = 1.085 p = 0.356	
**Stage**													
1	20	12.0	4.1	11.7	4.7	12.2	4.1	10.6	3.4	9.7	4.5	56.3	16.4
2	66	11.2	4.2	11.6	5.7	10.8	5.2	9.6	3.9	9.1	4.8	52.4	21.4
3	63	11.4	5.1	11.9	5.4	11.2	4.7	9.3	3.8	8.7	4.2	52.6	19.9
4	80	14.1	4.9	14.5	5.2	14.4	4.6	11.9	4.5	12.0	5.3	66.9	21.3
(Significance)		F = 6.115 p = 0.001		F = 4.839 p = 0.003		F = 9.132 p < 0.001		F = 7.303 p < 0.001		F = 6.837 p < 0.001		F = 8.852 p < 0.001	
**Surgery**													
No	125	13.8	4.6	14.4	5.1	13.8	4.7	11.5	4.2	11.7	4.9	65.3	20.2
Yes	131	11.4	4.8	11.6	5.5	11.3	4.9	9.6	3.9	8.7	4.6	52.8	20.9
(Significance)		F = 12.530 p < 0.001		F = 12.773 p < 0.001		F = 12.459 p < 0.001		F = 11.206 p = 0.001		F = 20.389 p < 0.001		F = 18.315 p < 0.001	
**Chemotherapy**													
No	109	13.7	5.0	14.3	5.5	13.6	5.1	11.6	4.3	10.8	5.3	64.1	22.2
Yes	147	11.8	4.6	12.0	5.3	11.7	4.8	9.7	3.9	9.8	4.7	55.1	20.2
(Significance)		F = 9.117 p = 0.003		F = 10.842 p = 0.001		F = 8.040 p = 0.005		F = 14.630 p < 0.001		F = 2.680 p = 0.103		F = 11.026 p = 0.001	

*M, mean; SD, standard deviation.*

### Correlation Between Multidimensional Fatigue Inventory Scales and the European Organization for Research and Treatment of Cancer Quality of Life Questionnaire Scales

Table 4 portrays the correlation between the five dimensions and the total score of the MFI-20, and the scales and the total score of the EORTC QLQ-C30. All the possible associations were found to be statistically significant (*p* < 0.001) with the exception of diarrhea. The strongest associations were found between general fatigue of the MFI-20 and the fatigue scale of the EORTC QLQ-C30 (*r* = 0.75), the MFI-20 total score and the fatigue scale of the EORTC QLQ-C30 (*r* = 0.74), and the MFI-20 total score and the EORTC QLQ-C30 sum score (*r* = −0.73). Stronger associations were also observed between the MFI-20 total score and the EORTC QLQ-C30 pain scale (*r* = 0.69), the general fatigue score of the MFI-20 and the EORTC QLQ-C30 sum score (*r* = −0.69), and the reduced activity score of the MFI-20 and the EORTC QLQ-C30 fatigue scale (*r* = 0.68).

**TABLE 4:** Correlations between the MFI-20 scales and the EORTC QLQ-C30 scales.

EORTC QLQ-C30	MFI-20					
	General fatigue	Physical fatigue	Reduced activity	Reduced motivation	Mental fatigue	Total score
Physical functioning	−0.63***	−0.62***	−0.64***	−0.48***	−0.55***	−0.67***
Role functioning	−0.66***	−0.61***	−0.62***	−0.48***	−0.50***	−0.66***
Emotional functioning	−0.54***	−0.48***	−0.47***	−0.43***	−0.54***	−0.56***
Cognitive functioning	−0.54***	−0.51***	−0.52***	−0.49***	−0.61***	−0.61***
Social functioning	−0.52***	−0.54***	−0.52***	−0.47***	−0.44***	−0.57***
Global health/QoL	−0.54***	−0.49***	−0.46***	−0.44***	−0.49***	−0.56***
**Fatigue**	**0.75** ***	**0.66** ***	**0.68** ***	**0.54** ***	**0.59** ***	**0.74** ***
Nausea/Vomiting	0.29***	0.27***	0.33***	0.26***	0.29***	0.33***
Pain	0.67***	0.65***	0.64***	0.48***	0.59***	0.69***
Dyspnea	0.36***	0.36***	0.37***	0.26***	0.35***	0.39***
Insomnia	0.42***	0.37***	0.39***	0.33***	0.46***	0.45***
Appetite loss	0.43***	0.44***	0.45***	0.30***	0.41***	0.47***
Constipation	0.27***	0.22***	0.23***	0.23***	0.24***	0.27***
Diarrhea	0.12*ns*	0.10*ns*	0.10*ns*	0.03*ns*	0.15*ns*	0.12*ns*
Financial difficulties	0.38***	0.36***	0.34***	0.29***	0.32***	0.39***
Sum score	−0.69***	−0.66***	−0.67***	−0.53***	−0.64***	−0.73***

***p < 0.001. ns, not significant, bold, correlations between the EORTC QLQ-C30 fatigue scale and the MFI-20 scales.

## Discussion

One of the objectives of this work was to examine the internal consistency of the MFI-20. Accordingly, the Cronbach’s alpha coefficient of the MFI-20 sum score of our Ethiopian patients with cancer (α = 0.93) was very good and comparable with the German sample (α = 0.94). Internal consistency of the MFI-20 scales in the Ethiopian sample was good (α ≥ 0.75), except for the reduced motivation scale (α = 0.60). This is similar to the studies with Indian patients with cancer (Chandel et al., 2015) and the Chinese general population (Chuang et al., 2018). The relatively lower degree of internal consistency on reduced motivation in our study could be accounted for by the lower educational status of the Ethiopian patients with cancer where 52.7% were illiterate. Yet, the coefficient in our study was better than in a Korean study (α = 0.52) on this dimension (Song et al., 2018).

The Pearson correlations between the MFI-20 and the EORTC QLQ-C30 sum scores and their respective scales were found to be statistically significant and in the expected directions. All the MFI-20 dimensions were negatively correlated with all the functioning scales and positively associated with all the symptom scales of the EORTC QLQ-C30. Of these, our work revealed strong association of the MFI-20 total score with the EORTC QLQ-C30 sum score (*r* = −0.73) and its fatigue scale (*r* = 0.74). Similarly, the general fatigue dimension of the MFI-20 was strongly associated with the fatigue scale of the EORTC QLQ-C30 (*r* = 0.75). The association between reduced activity of the MFI-20 and the fatigue scale of the EORTC QLQ-C30 was also strong (*r* = 0.68). Our work, therefore, shows a good convergent validity of the MFI-20, as applied to the Ethiopian patients with cancer. A study on the validation of the brief fatigue inventory among Ethiopian patients with cancer (Gebremariam et al., 2018) also reported a strong correlation between the scale’s total score and the EORTC QLQ-C30 fatigue scale (*r* = 0.75). When the association of general fatigue and that of the fatigue scale of EORTC QLQ-C30 alone was compared with a German study (Vollrath et al., 2013), the correlation in our study appeared to be higher (*r* = 0.60). The association between the MFI-20 dimensions and the EORTC QLQ-C30 fatigue scale in our work was also in line with a recent French study on QoL and fatigue as predictors of return to work during treatment among patients with breast cancer (Porro et al., 2019). In this longitudinal study, the correlation between the general and the physical fatigue of MFI-20 and that of the EORTC QLQ-C30 fatigue scale ranged from 0.60 to 0.72 before and after 6 months. However, this study did not report the MFI-20 sum score.

Mean score comparisons between our Ethiopian sample and the German sample showed that the burden of fatigue is generally higher in Ethiopian patients with cancer. The strongest difference was observed on the reduced motivation dimension (*d* = 0.46). This may be related to the multifaceted problems a recent qualitative study in Ethiopia has identified. The problems that may contribute to reduced motivation include challenges that are customer-related, provider-related, facility-related, and technology-related (Haileselassie et al., 2019), and all, in one way or the other, affect the patient’s motivation.

Analysis of variance was performed on socio-demographic and clinical characteristics of our Ethiopian patients with cancer considering gender and age as cofactors. Men reported higher mean scores than women. However, the difference was not statistically significant. Older patients with cancer reported a statistically significant (*p* = 0.011) higher mean score on reduced motivation compared with their younger counterparts. Such a difference was also observed between the two groups in the total score and the other dimensions of the MFI-20. However, none were statistically significant. In a Saudi Arabian study, younger patients with breast cancer reported higher mean scores on the functioning scale of the EORTC QLQ-30 (Imran et al., 2019). One can, therefore, conclude that older patients demonstrate higher mean scores on symptom scales, including fatigue.

Illiterate Ethiopian patients with cancer reported significantly higher MFI-20 mean sum scores in all the dimensions except in reduced activity when compared with the literate patients. Previous studies in Ethiopia reported that more than half the patients with cancer were illiterate (Ayana et al., 2018; Gebremariam et al., 2018; Araya et al., 2019; Haileselassie et al., 2019), which is comparable with the present work. One distinct characteristic of illiterate patients in the Ethiopian context is that they mostly reside in rural areas where life depends hugely on subsistence agriculture or pity trade. Living with cancer under such a life condition is more draining and exhausting. This might have, therefore, contributed to the elevated mean scores of fatigue on the part of the illiterate groups. A further possible reason for the high fatigue levels in illiterate patients is that they had difficulties to understand and interpret the questions and the answer options.

Our work revealed a linear and strong association between fatigue and tumor stage, where patients with stage 4 reported a significantly higher level of fatigue (*p* < 0.001). Previous studies have also identified fatigue as the most important symptom of advanced cancer (Butt et al., 2008). Patients with cancer who received surgery and chemotherapy reported a lower degree of fatigue than those who did not receive these treatments. However, this is not supported by other studies. For instance, an Indian study on fatigue and functional ability in patients with cancer undergoing surgery reported that postoperative fatigue levels were significantly higher compared with the preoperative levels (Parveen et al., 2019). Our findings may be related with the overall financial difficulties that were significantly associated with fatigue (*r* = 0.39; *p* < 0.001). It is also highly likely that in patients who do not receive surgery or chemotherapy it might be due to their inability to afford for such interventions.

There are limitations associated with this work. Participants were recruited from a single hospital; therefore, the results are not generalizable to national or regional levels. More than half the patients were illiterate and hence the nurse research assistants had to read every item aloud and complete the questionnaire for them. Although this is a common practice in low-income countries where illiteracy is still high, there is no evidence as to what extent this may affect the reliability and validity of such a study. A further problem is that the comparison group form Germany cannot be considered to be representative of patients with cancer in the western populations in general.

## Conclusion

The MFI-20 was found to be fairly applicable in the Ethiopian patients with cancer speaking Amharic regardless of their level of education. The difference between the Ethiopian and German patients with cancer on cancer-related fatigue demonstrates that cancer-related care and support in low-income countries needs to be addressed.

## Data Availability Statement

The raw data supporting the conclusions of this article will be made available by the authors, without undue reservation.

## Ethics Statement

The studies involving human participants were reviewed and approved by the Institutional Ethical Review Board of the University of Gondar, Ethiopia. The patients/participants provided their written informed consent to participate in this study.

## Author Contributions

YW and AH designed the study. YW recruited patients and obtained data. AH performed the statistical analysis. Both authors contributed to the article and approved the submitted version.

## Conflict of Interest

The authors declare that the research was conducted in the absence of any commercial or financial relationships that could be construed as a potential conflict of interest.

## Publisher’s Note

All claims expressed in this article are solely those of the authors and do not necessarily represent those of their affiliated organizations, or those of the publisher, the editors and the reviewers. Any product that may be evaluated in this article, or claim that may be made by its manufacturer, is not guaranteed or endorsed by the publisher.
